# Diagnostic Challenge of a Deep Minor Salivary Gland Neoplasm

**DOI:** 10.1155/2014/608267

**Published:** 2014-06-09

**Authors:** Vivian P. Wagner, Manoela D. Martins, Bruna Genari, Fernando B. do Amaral, Antônio C. Maciel, Marco A. T. Martins, Maria C. Munerato

**Affiliations:** ^1^Department of Oral Pathology, Dental School, Universidade Federal do Rio Grande do Sul, Ramiro Barcelos 2492, 90035-003 Porto Alegre, RS, Brazil; ^2^Department of Oral Medicine, Hospital de Clínicas de Porto Alegre (HCPA/UFRGS), Ramiro Barcelos 2350, 90035-903 Porto Alegre, RS, Brazil; ^3^Department of Otolaryngology-Head and Neck Surgery, Hospital de Clínicas de Porto Alegre (HCPA/UFRGS), Ramiro Barcelos 2350, 90035-903 Porto Alegre, RS, Brazil; ^4^Departement of Radiology, Hospital de Clínicas de Porto Alegre (HCPA/UFRGS), Ramiro Barcelos 2350, 90035-903 Porto Alegre, RS, Brazil

## Abstract

Core needle biopsy represents a safe and cheap alternative diagnostic method to open biopsy and fine-needle aspiration cytology in head and neck tumors. There is little evidence in the literature about the use of core needle biopsy in minor salivary gland lesions. This single case report presents a 60-year-old woman with a painless swelling in the soft palate, breathing and swallowing difficulties, and a feeling of suffocation. Two open biopsies had inconclusive diagnosis and the lesion could only be assessed and diagnosed as pleomorphic adenoma through core needle biopsy. Recognizing the correct indication of core needle biopsy can benefit both health professionals and patients; thus, it is important to consider the possibility of performing this method to diagnose minor salivary gland tumors.

## 1. Introduction

Salivary gland tumors are unusual oral conditions that generated considerable interest due to their heterogeneous histology, grade of malignancy, and clinical behavior. An accurate preoperative diagnosis is necessary to establish an adequate treatment and open biopsy followed by histopathological analysis is considered to be the gold standard for diagnosing salivary gland tumors [1]. This technique usually provides sufficient material for diagnosis; however, it represents an invasive method, might involve general anesthesia and hospital admission, and can be time consuming [2]. Procedures such as fine-needle aspiration cytology (FNAC) and core needle biopsy (CNB) have gained widespread popularity for tissue sampling in order to achieve a definitive diagnosis as they both represent less invasive and inexpensive techniques [3, 4]. FNAC is well established as a safe diagnostic method in head and neck tumors, but it presents some disadvantages such as the elevated number of nondiagnostic samples and requirement of experienced cytologist to assure a correct diagnosis [2].

CNB appears as a good alternative to open biopsy and FNAC, as it comprises safety, low cost, and low invasiveness with the advantage of offering suitable tissue sample for histopathological and immunohistochemical analysis [5]. The advantages of using CNB in head and neck tumors have already been demonstrated [2, 4, 6], including lesions located in major salivary glands [5, 7]; however, no study was found discussing the application of CNB in minor salivary glands. This paper presents a case of pleomorphic adenoma located on the soft palate wherein the diagnosis was obtained using CNB following two inconclusive conventional biopsies. A discussion of the importance of this technique in the diagnosis of salivary gland tumors is also offered.

## 2. Case Presentation

**Figure 1:**
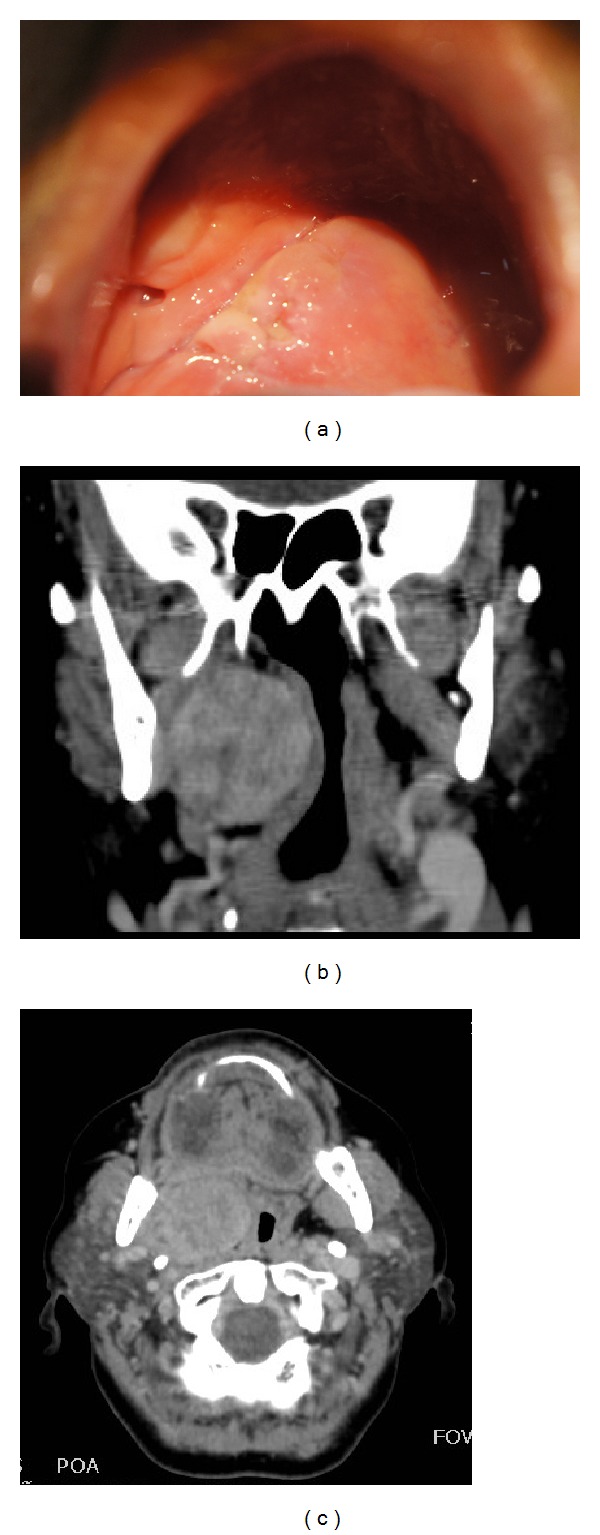
Nodular lesion on right side of soft palate lined by normal mucosa (a). Axial (b) and coronal (c) computed tomography scans showing a hyperdense expansive lesion with well-defined limits measuring 3.7 × 3.3 cm, determining compression and displacement to the left part of the nasopharynx and oropharynx, and causing narrowing of the airways.

**Figure 2:**
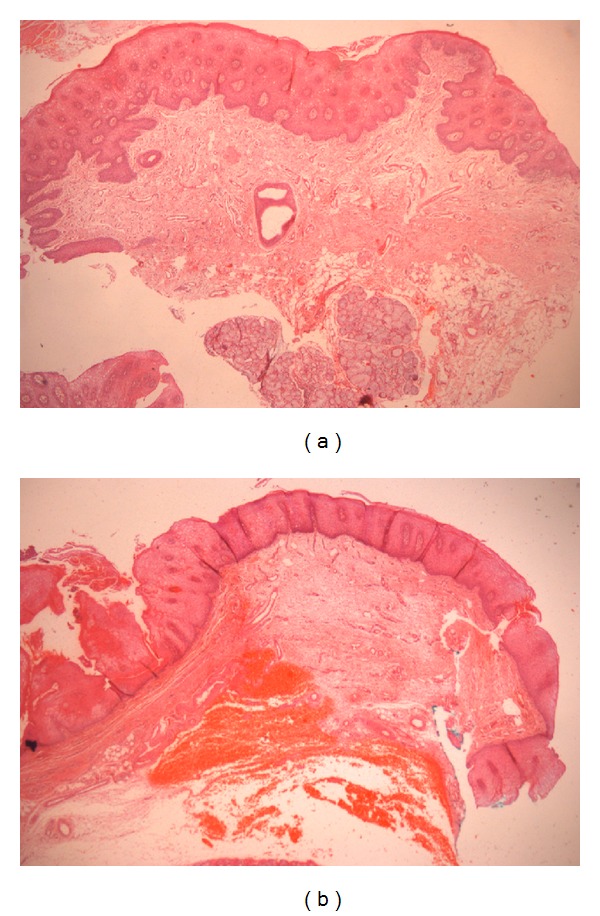
Histopathological aspects of previous biopsies with conventional technique showing epithelial tissue, connective tissue, and minor adjacent salivary glands, with no sign of tumor (H&E ×40) (a, b).

A 60-year-old Caucasian female patient presented with a painless swelling in the soft palate, breathing and swallowing difficulties, and suffocation feeling. Clinical examination revealed that the lesion was located mostly in the right side, extending from the limit between the hard and soft palate and continuing to the oropharynx. The lesion had smooth surface, hard consistency, and irregular shape (Figure 1(a)). Cervical tomography was requested (Figures 1(b) and 1(c)). Based on clinical and imaging aspects, a hypothesis of benign X malignant salivary gland tumor was formulated. An incisional biopsy was performed under local anesthesia to establish the definitive diagnosis. However, the histopathological examination of the fragment revealed normal mucosa (Figure 2(a)). The professionals decided to perform a second conventional biopsy giving attention to collecting material from a greater depth. Once again, the histopathological analysis revealed normal mucosa (Figure 2(b)).

**Figure 3:**
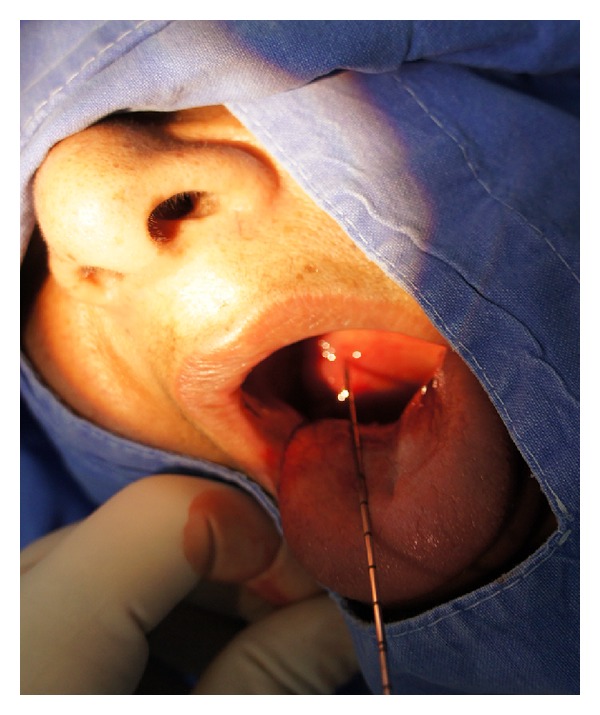
CNB procedure under local anesthesia.

**Figure 4:**
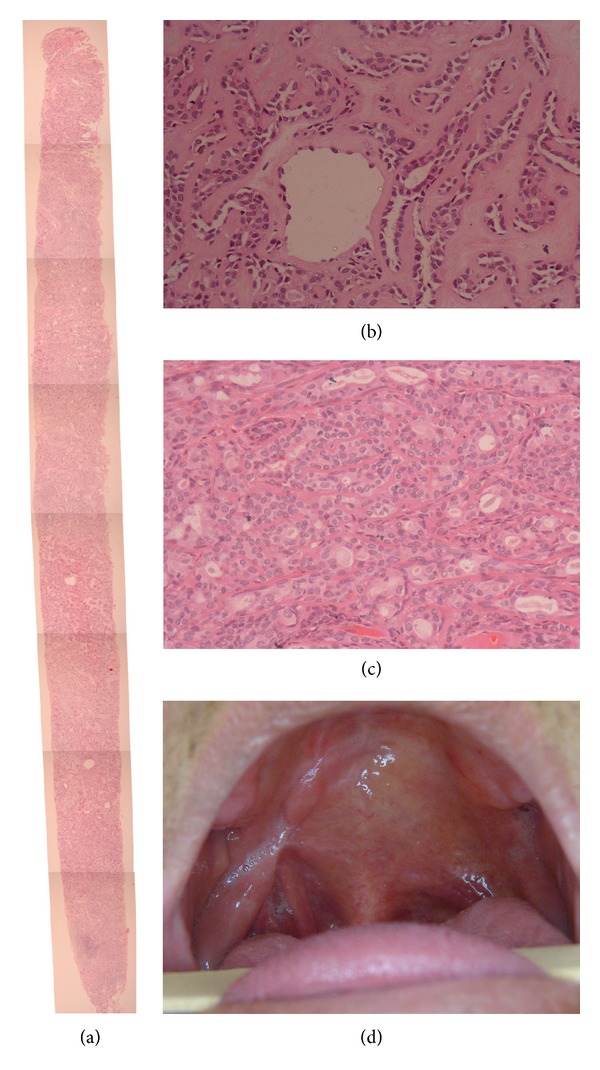
Histopathological aspects of entire fragment obtained after CNB (H&E ×40) (a) Strands and sheets of epithelial cells with ductal structures. Deposition of homogeneous, eosinophilic, hyaline material between tumor cells and dispersed in extracellular matrix (H&E ×100) (b) Histopathological aspects of surgical specimen confirming diagnosis of pleomorphic adenoma (H&E ×100) (c). Intraoral aspect at 12-month follow-up, with no signs of recurrence (d).

The otorhinolaryngologist team was consulted and the decision was made to perform a CNB. The localization of the lesion was identified during the intraoral exam with no need of imaging guidance (Figure 3). In CNB a needle is activated by an automatic spring system equipped with outer cannula and inner stylet. Under local anesthesia using 1% lidocaine the biopsy area was chosen with palpation of the lump and the tip of the needle was positioned adjacently to the lesion. The handpiece was activated, moving quickly 20 mm back and forth, cutting a sample measuring 17 mm in length. Only one core of tissue was obtained and carefully removed from the biopsy needle. The quality of the specimen was assessed by visual inspection and, with care not to damage the thin tissue, it was put into 10% formalin for fixation. The biopsy site was compressed for five minutes to avoid bleeding and the patient was observed for more 30 minutes and later discharged as no signs or symptoms as bleeding or pain were noted. The histopathological analysis of the sample revealed pleomorphic adenoma (Figures 4(a) and 4(b)).

The patient was referred to the head and neck service for complete resection of the tumor. The specimen was sent to the pathology service and the histopathological analysis confirmed the diagnosis established with CNB (Figure 4(c)). No complications occurred in the postoperative period and no signs of recurrence were found at the 12-month follow-up (Figure 4(d)).

## 3. Discussion

Salivary gland tumors are the most heterogeneous group of tumors of the upper aerodigestive tract. Clinical examination and imaging exams cannot distinguish between benign and malignant tumors, and diagnosing these lesions can be extremely challenging. The probability of a salivary gland malignancy increases in inverse proportion to the size of the gland. Thus, unlike major salivary gland tumors, the majority of minor salivary gland tumors are malignant. As some cases considered benign could actually be misdiagnosed salivary gland carcinomas, the precise preoperative diagnosis of salivary gland tumors is necessary for appropriate patient management and surgical planning [8].

Open biopsy is generally considered the gold standard for obtaining a final diagnosis and usually provides sufficient material for achieving a diagnosis; however, it is invasive and in some cases involves general anesthesia and hospital admission and is time consuming [2]. In recent years, alternative procedures for preoperative assessments, like FNAC and CNB, have been increasingly used as an alternative approach for appropriate management decisions [3–5]. In some cases, especially in major salivary glands, open biopsy is no longer justified due to the high risk of tumor seeding, facial nerve injury, facial scarring, and fistula formation [5]. FNAC appears as an established safe and inexpensive method in the diagnosis of head and neck lesions; however, disadvantages of this technique include a high rate of nondiagnostic samples and false-negative results. Furthermore, FNAC should be performed and examined by an experienced cytologist to assure a satisfactory specimen and a correct diagnosis [2].

CNB principle of indication is to reach deep spaces with minimum violation of the superficial tissue layers, thus decreasing patients' morbidity while allowing a great diagnostic yield [6]. Based on this indication, CNB is broadly used by mastologists for the diagnosis of breast lumps and also used to achieve deeper organs like liver and lumps. In head and neck tumors, this procedure is less frequently used but can bring several benefits when correctly applied. When CNB and FNAC were compared for the capacity of supplying a specific diagnose in head and neck tumors, CNB presented 90% of correct estimated cases while FNAC showed only 66%; besides FNAC had to be repeated in 32% of the procedures due to initial nondiagnostic material [4]. When diagnostic accuracies of CNB and FNAC were compared particularly in parotid masses, CNB proved to have significantly higher sensitivity and overall accuracy and provided more specific diagnosis than FNAC [3].

In CNB, a needle is activated by an automatic spring system equipped with an outer cannula and an inner stylet; thus, this technique is able to supply a tissue sample wherein the lesion's histologic architecture is preserved enabling histopathological and immunohistochemical exams [2, 5]. A meta-analysis and systematic review of the literature tested the sensitivity, specificity, accuracy, and the positive and negative predictive values of CNB in the identification of true neoplasms and detection of malignancy head and neck lesions. The study showed that CNB was an excellent method in the assessment of salivary gland lesions, with a positive predictive value of 100% for identification of true salivary glands neoplasms and of 98% in the identification of malignancy in such lesions [6]. All studies included in this review only presented cases of major salivary gland lesions, especially located in the parotid gland.

Although usually minor salivary glands allow easy access to carry out incisional biopsies, there may be cases of false-negatives, as in the case described herein. This occurs in cases where the tumor is situated deep in the submucosa with a large band of connective tissue covering and separating it from the mucosa surface. In these cases it is important that clinicians be aware of CNB as an important diagnostic tool and know its efficiency in the diagnosis of these lesions. Malignant and benign tumors of minor salivary glands have similar clinical aspects and it is important to achieve a final diagnosis to indicate the correct treatment for each case.

A study conducted on 27 lesions located in the parotid gland and on 10 in the submandibular gland found a sensitivity of 75.0% and specificity of 96,6% for CNB as a diagnostic method [7]. It is important to highlight that there were no samples in the nondiagnostic category [7], a problem that is usually found with FNAC [6]. CNB can raise some difficulties in getting a representative histology sample from cystic lesions and small tumors (less than 1 cm) [3]. When a biopsy imaging guidance is used, smaller and impalpable lesions can be approached [7]. The ultrasound appears as a good alternative because of its ease of use and real-time capability, as it assures the safety of the biopsy [3]. As CNB does not allow aspiration of cystic content, FNAC should be preferably used when cystic lesions are suspected [4].

CNB associates the advantages of a cheap and minimal invasive technique, such as FNAC, with the possibility of obtaining a core of tissue passable for histological and immunohistochemical analyses, reducing the possibility of a nondiagnostic sample [2, 5]. In the present study, CNB was not used for early diagnosis since conventional biopsy is generally employed for superficial tumors in the minor salivary glands. However, in the case described herein, the tumor exhibited expansive growth and was situated in the deep portion of the tissue, making it difficult for conventional scalpel use to access the tumor and establish the diagnosis. Previous studies have demonstrated the advantages of the use of CNB in head and neck tumors [2, 4–7]; however, no study was found discussing the application of CNB in minor salivary glands. It is important to highlight that CNB diagnosis of pleomorphic adenoma was confirmed in the surgical specimen histological analysis. Frequently, differentiation between malignant and benign salivary gland tumors is predicated on the presence or absence of invasion; thus, the histological examination of tumor's periphery is of paramount importance to determine whether infiltrative growth is or is not present. This pitfall, however, is present for all diagnostic strategies including open biopsy, CNB, and FNAC. Therefore it is important to emphasize that a definitive diagnosis requires a careful evaluation of the surgical specimen [9].

Recognizing the correct indication of CNB can benefit both health professionals and patients. Open biopsy remains the gold standard for minor salivary gland lesions; however, the purpose of this case was to demonstrate that this alternative method might be useful for the diagnosis of tumors situated in greater depth of tissues. CNB represents a safe technique and at the present case was able to supply a correct diagnose, confirmed in the surgical specimen.
